# Appropriateness of Prescribing Rivaroxaban at King Khalid University Hospital Riyadh

**DOI:** 10.7759/cureus.20977

**Published:** 2022-01-05

**Authors:** Ghazi I Arishi, Mohammed S Sheik, Abdulaziz Alhossan

**Affiliations:** 1 Pharmaceutical Care Services, Prince Muhammed Bin Nassir Hospital, Ministry of Health, Jazan, SAU; 2 Pharmaceutical Care Services, Attwal General Hospital, Ministry of Health, Jazan, SAU; 3 College of Pharmacy, Riyadh Elm University, Riyadh, SAU; 4 College of Pharmacy, King Saud University, Riyadh, SAU

**Keywords:** atrial fibrillation management, direct oral anticoagulant therapy, rivaroxaban, apixaban, anticoagulant therapy

## Abstract

Background and aim

Warfarin is recognized as a first-line treatment for different coagulopathy conditions; however, guidelines also encourage the use of rivaroxaban as an alternative option. The recent approval of the novel oral anticoagulants (NOACs) has led to swift changes in anticoagulant prescribing practices. This study aimed to review rivaroxaban prescribing patterns in adult patients in a large tertiary care setting in the Kingdom of Saudi Arabia (KSA).

Materials and methods

A retrospective cross-sectional study was conducted from January 2019 to September 2020 at King Khalid University Hospital, Riyadh, KSA. Data was collected from the patient's medical record. Data analysis was performed with the Statistical Package for the Social Sciences (SPSS) IBM Corp. Released 2013. IBM SPSS Statistics for Windows, Version 22.0. Armonk, NY: IBM Corp.

Results

A total of 309 patients were included in this study. Rivaroxaban use for non-valvular atrial fibrillation (NVAF) was relatively higher than deep venous thrombosis/pulmonary embolism (DVT/PE). 45% of the patients had NVAF, followed by DVT/PE (26%), and DVT/PE prophylaxis (25%). Fifty-six patients, (18%) received an inappropriate dose of rivaroxaban for NVAF.

Conclusion

This study found a relatively high percentage of inappropriate rivaroxaban prescribing, predominantly because of inappropriate dosing, which can potentially increase medication-related events. The use of rivaroxaban should be monitored to increase the appropriateness of therapy and improve patient safety.

## Introduction

Anticoagulant therapy is the cornerstone of venous thromboembolism (VTE) treatment [1]. For more than 50 years, warfarin was the only available option as an oral anticoagulant (OAC) for the treatment of atrial fibrillation and other thrombotic conditions [2]. The use of warfarin has also been linked with many safety reports and associated with several adverse events during routine clinical practice [3-5]. Novel oral anticoagulants (NOACs) or direct oral anticoagulants (DOACs) such as rivaroxaban, edoxaban, and apixaban are now approved factor Xa inhibitors that provide anticoagulation via oral route [6]. These NOACs have expanded the options of OACs available to healthcare professionals. NOACs are attractive treatment options due to numerous challenges with warfarin therapy such as frequent monitoring, drug interactions, delayed time to onset, and a narrow therapeutic index [7]. In 2011, rivaroxaban was initially approved by the US. Food and Drug Administration (FDA) in knee and hip replacement surgery patients for preventing deep venous thrombosis (DVT). In the same year, it was approved for the prevention of stroke and systemic embolism in non-valvular atrial fibrillation (NVAF). Furthermore, in 2012, rivaroxaban was also approved for the treatment of DVT and pulmonary embolism (PE) and for the risk reduction of recurrent DVT and PE [8].

An open-label and randomized clinical trial concluded that rivaroxaban offers a simple, single-drug approach to the short-term and continued treatment of venous thrombosis that may improve the benefit-to-risk profile of anticoagulation [9]. A comparative retrospective study revealed that the effectiveness and risks of rivaroxaban versus warfarin varied by prior anticoagulant status, whereas the effectiveness of rivaroxaban versus dabigatran varied in gastrointestinal (GI) bleeding risk [10]. A retrospective cohort study in Canada found that the incidence of inappropriate use was 26.9% for rivaroxaban. The most common reason for inappropriate use was prescribed for an unapproved indication [11].

Guidelines encourage the use of rivaroxaban as an alternative option for the prevention of stroke and embolism and VTE prophylaxis [12-14]. Since its addition to the hospital formulary, it is imperative to monitor the appropriateness of rivaroxaban as the large number of patients in our study setting has received this drug for the prevention, treatment, and reducing the risk of multiple diseases. Little is known about the appropriateness of rivaroxaban in the tertiary care settings of the Kingdom of Saudi Arabia (KSA). Therefore, this study aims to review rivaroxaban prescribing patterns in adult patients in a large tertiary care setting in the KSA.

## Materials and methods

A retrospective cross-sectional study was conducted from January 2019 to September 2020. The study took place in King Khalid University Hospital (KKUH), a tertiary care setting with an 850-bed capacity in Riyadh, KSA. This hospital provides free medical services to eligible patients and serves a wide range of patients drawn from a large population, many of whom present with complex medical comorbidities and are referred from different regions of the KSA.

The data was collected from the cardiology and hematology department and included patient age, gender, rivaroxaban indication, dose, frequency, prescriber specialty, use of acetylsalicylic acid, non-steroidal anti-inflammatory drug (NSAID) or antiplatelet agent, patient’s weight, height, serum creatinine, and creatinine clearance (ml/min). A data collection sheet was designed to collect the study data. This study was initiated after approval from the ethics committee of KKUH (Reference number: E-20-6004). A waiver of written informed consent was granted by the ethics committee.

All patients aged 18 years and over who received rivaroxaban between January 2019 and September 2020 were enrolled in the study. Data analysis was performed with the Statistical Package for the Social Sciences (SPSS) software, (IBM Corp. Released 2013. IBM SPSS Statistics for Windows, Version 22.0. Armonk, NY: IBM Corp.). Age, weight, height, body mass index (BMI), serum creatinine, and creatinine clearance variables were presented in mean ±standard deviation. Median and range used for categorical study variables such as gender, indications, dose, frequency, prescribing department, and concomitant medications were described in frequencies and percentages. Stratification was done for age, BMI, creatinine clearance for univariate analysis. Analysis of variance (ANOVA) for mean difference and Chi-square test for association was applied accordingly. A p-value of less than 0.05 was considered statistically significant.

## Results

A total of 309 patients on rivaroxaban were included in this study. All patients came from both cardiology, hematology departments. The mean age was 62.2 years (±18.1) and approximately 64% of the study participants were female. The mean weight and height of the patients were 79.7 kg (±19.6) and 160.2 cm (±9.2) respectively. The mean creatinine clearance was 100.9 (±55.7) (Table 1).

**Table 1:** Description of demographics and lab investigations in study patients BMI: body mass index, SD: standard deviation. All numerical data are presented in mean ±SD. All categorical data are presented in n (%).

Variables	Overall (n=309)
Age, years	62.2 ±18.1
Gender	
Male	112 (36.2%)
Female	198 (63.8%)
Weight, kg (Mean ±SD)	79.7 ±19.6
Height, cm (Mean ±SD)	160.2 ±9.2
BMI, kg/m^2 ^(Mean ±SD)	30.9 ±6.9
Serum Creatinine, mmol/L (Mean ±SD)	83.2 ±46.3
Creatinine Clearance, mL/min (Mean ±SD)	100.9 ±55.7

**Figure 1:**
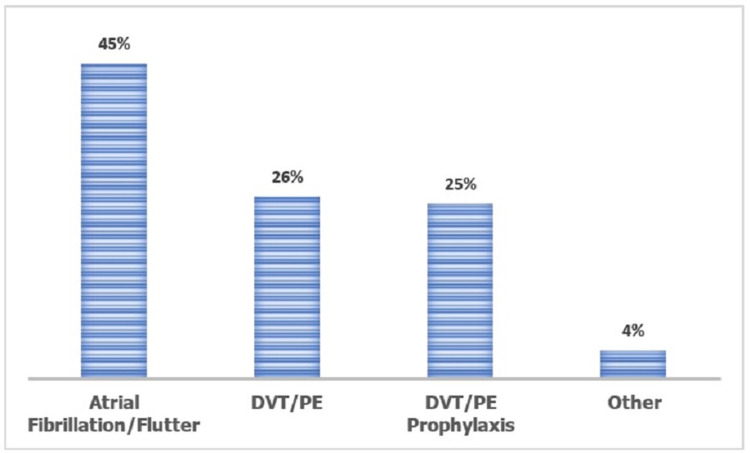
Percentage of indications for rivaroxaban (n=309) DVT: Deep Vein Thrombosis, PE: Pulmonary Embolism.

Most of the prescriptions are using rivaroxaban for non-valvular atrial fibrillation 45% followed by DVT/PE (26%), and DVT/PE prophylaxis (25%) (Figure 1).

The most common dose of rivaroxaban was 10 mg (37.5%) followed by 15 mg (34%), and 20 mg (28.5%). Ninety-six percent of the patients received rivaroxaban once daily. Rivaroxaban was also given twice a day in 3.3% of patients. More than one-third of the prescriptions were prescribed in the cardiology department, followed by hematology (25.9%), and others (31.7%). Most of the patients (85.8%) were not taking any concomitant medications. The most common concomitant medications were aspirin, clopidogrel, warfarin and celecoxib (Table 2).

**Table 2:** Prescription characteristics All categorical data are presented in n (%).

Variables	Overall (n=309)
Dose	
10 mg	116 (37.5%)
15 mg	105 (34%)
20 mg	88 (28.5%)
Frequency	
Once daily Twice daily	296 (95.8%)
Twice daily	10 (3.3%)
Every other day	1 (0.3%)
Data missing	2 (0.6%)
Prescriber department	
Cardiology	131 (42.4%)
Hematology	80 (25.9%)
Anticoagulant	31 (10%)
Other	67 (21.7%)
Concomitant medications	
Yes	44 (14.2%)
No	265 (85.8%)
Details of Concomitant Medication (n=44)	
Warfarin	2 (4.5%)
Clopidogrel 75 mg	5 (11.4%)
Aspirin 81 mg	31 (70.5%)
Celecoxib 200 mg	3 (6.8%)
Topical Voltaren	3 (6.8%)

To assess the association of variables and rivaroxaban use we found that 56 patients aged between 60 and 79 years were on rivaroxaban for the treatment of NVAF. Likewise, 43 patients from the same age group received rivaroxaban as prophylaxis for DVT/PE. A majority (n=138) of the patients who were prescribed rivaroxaban for NVAF were females. Interestingly, only one male patient received rivaroxaban for NVAF. Based on the mean value of BMI, patients suffering from NVAF, and DVT/PE were obese. Nearly 80% (n=247) of the patients had a creatine clearance > 50 (Table 3). Nonetheless, there was no statistically significant difference (0.324) between the groups of creatine clearance and the indications of rivaroxaban (Table 3).

**Table 3:** Association of age, gender, BMI and creatinine clearance with indications of rivaroxaban NVAF: Non-valvular Atrial Fibrillation, DVT: Deep Vein Thrombosis, PE: Pulmonary Embolism, BMI: Body Mass Index, Cr.Cl: Creatinine Clearance, SD: Standard Deviation. P-value <0.05 was considered statistically significant.

Variables	NVAF	DVT/PE Treatment	DVT/PE Prophylaxis	Others	P-value
Age, years; mean ±SD	64.8 ±17.5	55.1 ±19.6	65.0 ±16.0	61.2 ±18.1	0.001
Age Groups; n (n%)					
19 – 39	13 (32.5%)	20 (50%)	6 (15%)	1 (2.5%)	0.006
40 – 59	40 (46%)	25 (28.7%)	17 (19.5%)	5 (5.7%)	
60 – 79	56 (43.8%)	24 (18.8%)	43 (33.6%)	5 (3.9%)	
≥ 80	30 (55.6%)	10 (18.5%)	12 (22.2%)	2 (3.7%)	
Gender					
Male	1 (0.9%)	60 (53.6%)	42 (37.5%)	9 (8%)	<0.001
Female	138 (70.1%)	19 (9.6%)	36 (18.3%)	4 (2%)	
BMI, kg/m^2^; mean ±SD	30.9 ±7.1	30.6 ±6.9	31.2 ±6.4	31.9 ±8.2	0.896
BMI Groups; n (n%)					
< 18.5	4 (66.7%)	2 (33.3%)	0	0	0.015
18.5 – 22.9	19 (65.6%)	5 (17.2%)	5 (17.2%)	0	
23 – 24.9	6 (20%)	14 (46.7%)	7 (23.3%)	3 (10%)	
≥ 25	104 (43.9%)	57 (24.1%)	66 (27.8%)	10 (4.2%)	
Cr.Cl. mL/min; mean ±SD	100.5 ±59.8	106.3 ±53.5	95.9 ±53.2	102.1 ±38.1	0.714
Cr.Cl. Groups; n (n%)					
< 30	9 (60%)	2 (13.3%)	3 (20%)	1 (6.7%)	0.324
30 – 50	22 (56.4%)	7 (17.9%)	10 (25.6%)	0	
> 50	103 (41.7%)	69 (27.9%)	63 (25.5%)	12 (4.9%)	

Table 4 shows the association of dose, frequency, department, and concomitant medication with indications of rivaroxaban.

**Table 4:** Association of dose, frequency, department & concomitant medication with indications of rivaroxaban NVAF: Non-valvular Atrial Fibrillation, PE: Pulmonary Embolism. P-value <0.05 was considered statistically significant

Variables	NVAF	DVT/PE Treatment	DVT/PE Prophylaxis	Others	P-value
Dose					
10 mg	56 (48.3%)	42 (36.2%)	12 (10.3%)	6 (5.2%)	<0.001
15 mg	62 (59%)	14 (13.3%)	24 (22.9%)	5 (4.8%)	
20 mg	21 (23.9%)	23 (26.1%)	42 (47.7%)	2 (2.3%)	
Frequency					
Once-daily	131 (44.3%)	78 (26.4%)	77 (26%)	10 (3.4%)	0.001
Twice daily	6 (60%)	1 (10%)	0	3 (30%)	
Every other day	1 (100%)	0	0	0	
Prescriber Department					
Cardiology	66 (50.4%)	20 (15.3%)	44 (33.6%)	1 (0.8%)	0.337
Haematology	28 (35%)	30 (37.5%)	14 (17.5%)	8 (10%)	
Anticoagulant	14 (45.2%)	10 (32.3%)	3 (9.7%)	4 (12.9%)	
Other	4 (50%)	2 (25%)	2 (25%)	0	
Unknown	27 (45.8%)	17 (28.8%)	15 (25.4%)	0	
Concomitant Medication					
Yes	23 (52.3%)	11 (25%)	8 (18.2%)	2 (4.5%)	0.650
No	116 (43.8%)	68 (25.7%)	70 (26.4%)	11 (4.2%)	

**Figure 2:**
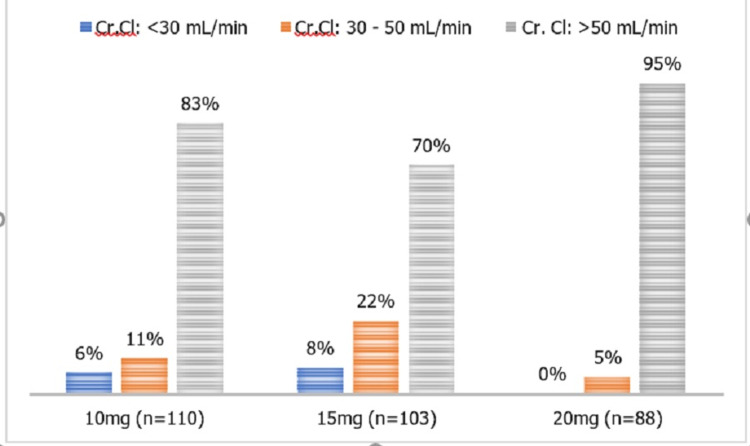
Percentage of patients who received rivaroxaban dose as per levels of creatinine clearance (n=301) Cr.Cl=Creatinine Clearance

Figure 2 depicts the percentage of patients who received rivaroxaban dose according to the level of creatinine clearance.

The reasons for inappropriate prescriptions among indications of rivaroxaban are presented in Table 5. The prescriptions of rivaroxaban were inappropriate in 56 patients (18%) in which the dose of rivaroxaban was incorrect for the indication of NVAF. Furthermore, 42 patients received inappropriate doses of rivaroxaban for the treatment of DVT/PE. Frequency of rivaroxaban and creatinine clearance were also the most common reasons for inappropriate rivaroxaban prescriptions. In addition, rivaroxaban was also prescribed inappropriately in patients with antiphospholipid syndrome APS (2%) (Table 5).

**Table 5:** Reasons of inappropriate prescriptions among indications of rivaroxaban Cr.Cl=Creatinine Clearance

Variables	Non-valvular Atrial Fibrillation (n=139)	Deep Vein Thrombosis/ Pulmonary Embolism Treatment (n=78)	Antiphospholipid syndrome
Inappropriateness due to dosing	56 (40%)	42 (53%)	-
Inappropriateness due to frequency	7 (5%)	54 (68%)	-
Inappropriateness based on Cr.Cl (Cr.Cl <30 mL/min)	9 (6%)	2 (3%)	-
Inappropriateness due to indications	-	-	8 (2%)

## Discussion

Monitoring of high-alert medications utilization is a very important aspect to prevent any possible adverse events as well as to optimize patient care. Recent approvals of the NOACs have led to swift changes in anticoagulant prescribing practices, in which these medications require a comprehensive knowledge of pharmacology, careful patient selection, and monitoring to ensure the best clinical outcomes [15].

This retrospective study identified a high proportion of inappropriate prescribing of rivaroxaban. This is consistent with the results from a similar study setting [16]. In our study, the most common reasons noted in rivaroxaban’s inappropriate prescribing were dosing, frequency, and indication. However, the percentage of the inappropriateness of rivaroxaban prescription based on creatinine clearance was low. Based on clinical guidelines, rivaroxaban is contraindicated in patients with creatinine clearance < 30 mL/min for a therapeutic dose [17]. The majority of the issues related to inappropriateness were noticed in dosing and frequency of rivaroxaban. Several patients in our study received 10 mg once daily dose of rivaroxaban for NVAF. Whereas the recommended dose for NVAF is 20 mg once daily [17]. Our findings related to the dosing concurs with the result from Whitworth et al. who also highlighted the matter of inappropriate rivaroxaban dosing in several hospitalized patients [18]. Simon et al. also reported similar findings related to the inappropriate dosing of rivaroxaban [19].

This study revealed that rivaroxaban was also prescribed along with other anticoagulants. Concomitant prescribing of the NOAC with an additional anticoagulant such as warfarin has serious potential of adverse events [15]. The possible reason behind that is probably the patient was switched to rivaroxaban without stopping the initial therapy with warfarin. Therefore, electronic systems with flagging features should be in place to ensure that whenever rivaroxaban or any other NOAC is being initiated for patients, other anticoagulation therapies should be stopped or overridden. In our study, rivaroxaban was also prescribed along with antiplatelet agents such as aspirin and clopidogrel in some patients. Concomitant administration of aspirin and clopidogrel with rivaroxaban is not recommended unless clinically indicated because dual therapy is associated with higher risks of bleeding and anemia, particularly in the presence of other risk factors [15]. In the future, there is a need to assess the impact of educational interventions on improving the appropriateness of rivaroxaban prescribing in a healthcare setting.

This study has some limitations, it was conducted in a single healthcare setting. Therefore, these findings should be validated by a multicentre longitudinal study across healthcare settings in the KSA.

## Conclusions

This study found a relatively high percentage of inappropriate rivaroxaban prescribing, predominantly because of incorrect dosing and inappropriate prescribing with concomitant anticoagulants, which can potentially increase medication-related events. The use of rivaroxaban should be monitored to increase the appropriateness of therapy and improve patient safety. Pharmacy-led initiatives and educational interventions, with priority given to the main factors identified as being associated with inappropriate use, are recommended at the study center to improve the safe use of this new anticoagulant and to prevent possible patient harm.
